# DNA methylation fingerprint of hepatocellular carcinoma from tissue and liquid biopsies

**DOI:** 10.1038/s41598-022-15058-0

**Published:** 2022-07-07

**Authors:** Emanuel Gonçalves, Maria Gonçalves-Reis, José B. Pereira-Leal, Joana Cardoso

**Affiliations:** 1 Ophiomics, Pólo Tecnológico de 8, R. Cupertino de Miranda 9, 1600-513 Lisbon, Portugal; 2 Present Address: INESC-ID, 1000-029 Lisbon, Portugal

**Keywords:** Computational biology and bioinformatics, High-throughput screening, Cancer genomics, Diagnostic markers, Cancer screening

## Abstract

Hepatocellular carcinoma (HCC) is amongst the cancers with highest mortality rates and is the most common malignancy of the liver. Early detection is vital to provide the best treatment possible and liquid biopsies combined with analysis of circulating tumour DNA methylation show great promise as a non-invasive approach for early cancer diagnosis and monitoring with low false negative rates. To identify reliable diagnostic biomarkers of early HCC, we performed a systematic analysis of multiple hepatocellular studies and datasets comprising > 1500 genome-wide DNA methylation arrays, to define a methylation signature predictive of HCC in both tissue and cell-free DNA liquid biopsy samples. Our machine learning pipeline identified differentially methylated regions in HCC, some associated with transcriptional repression of genes related with cancer progression, that benchmarked positively against independent methylation signatures. Combining our signature of 38 DNA methylation regions, we derived a HCC detection score which confirmed the utility of our approach by identifying in an independent dataset 96% of HCC tissue samples with a precision of 98%, and most importantly successfully separated cfDNA of tumour samples from healthy controls. Notably, our risk score could identify cell-free DNA samples from patients with other tumours, including colorectal cancer. Taken together, we propose a comprehensive HCC DNA methylation fingerprint and an associated risk score for detection of HCC from tissue and liquid biopsies.

## Introduction

Liver cancer is one of the deadliest types of cancer, with a 5-year overall survival rate lower than 20% and death rates increasing around 1.7% each year^1,2^. Hepatocellular carcinoma (HCC) is the most common malignancy of the liver accounting for nearly 90% of all cases^1,3–5^. Major risks of HCC include cirrhosis, viral infection with hepatitis B virus (HBV) or hepatitis C virus (HCV), alcoholic liver, non-alcoholic fatty liver disease and inherited traits such as metabolic diseases^1,6^. Current HCC diagnostic guidelines report the usage of invasive procedures, such as tissue biopsies, followed by histological and/or contrast-enhanced imaging^7^. This contributes to HCC being often detected in an advanced stage where it is estimated that 40% of the cases are multinodular or expanded beyond the liver leaving patients with limited therapeutic options^5,8^. Screening, surveillance and monitoring programmes are therefore vital to diagnose and detect HCC as early as possible to provide patients with the best treatment possible^9–11^. In addition, HCC patients surgically treated often experience relapses and early detection could bring better management of the disease and increase patient’s life quality and span^12^.

Body fluids, for example plasma, serum and urine, contain circulating biomarkers that can be measured non-invasively and inexpensively for diagnosis and monitoring of HCC^5,13,14^. Among others, alpha-fetoprotein (AFP) is often proposed as a diagnostic biomarker present in serum or plasma of high-risk individuals for HCC^7,13,15^, nonetheless official guidelines indicate that AFP has no diagnostic approved role^3,4^. High levels of AFP are considered diagnostic of HCC with almost perfect specificity, although sensitivity (recall) rates are frequently low, less than 45%^7^. Lower thresholds of AFP (20 ng/ml) comprises a balance between specificity and sensitivity with both ranging around 79%^7^. Of note, in patients with chronic liver disease, the population where screening methods are most needed, the precision of AFP is significantly reduced and insufficient for robust diagnosis^7,16,17^. This is particularly problematic since chronic liver diseases are the major risk factor for HCC, thus novel non-invasive and accurate clinical approaches are needed to improve cancer detection.

Liquid Biopsies (LBs) have recently emerged as a promising approach for early detection of tumours by characterising circulating tumour cells or circulating tumour free nucleic acids^18^. LBs contain cell-free DNA (cfDNA) material evocative of cells from the entire body, including varying levels of circulating tumour DNA (ctDNA)^19,20^ that is estimated to range between 0.1% and 10% in cancer patients^21–23^. Measurement of genetic markers in ctDNA, such as mutations and methylation, can be used as a diagnostic and therapeutic tool^13,18,20,24–32^ and provide complementary information to tissue samples, for example circumventing potential tissue heterogeneity which might result in sampling bias^33,34^.

DNA methylation plays an important role in cancer initiation and progression through the repression of tumour suppressor genes by promoter hypermethylation and promoter hypomethylation of many oncogenes^35–38^. Importantly, DNA methylation changes characteristic of cancer cell formation are often observed in early stages of carcinogenesis^39–42^. Hence, ctDNA methylation holds great promise for early cancer detection and monitoring, with systematic studies showing it outperforms other genetic markers like mutations and copy number alterations^26,43^. For example, promoter methylation of the gene Septin 9 (SEPT9/ SEPTIN9) is a plasma derived biomarker for colorectal cancer and is being studied for HCC^27,44^. Several studies have focused on the identification of DNA methylation biomarkers for HCC^43,45–48^, nonetheless these were limited to either tissue samples only, focused on the identification of small sets of single CpG sites, and/or mostly compared to healthy liver tissue samples. Relying on the accurate measurement of very specific and small sets of methylation biomarkers, mostly derived from tissue samples, may hinder the clinical generalisation of these methylation signatures to LBs and other cohorts. Additionally, it is fundamental to ensure that signatures can distinguish HCC patients from a background of chronic liver diseases, where current non-invasive molecular markers perform worse^7,16,17^.

Here, we perform a systematic discovery of a HCC methylation signature by compiling 1551 genome-wide DNA methylation arrays from 13 studies^1,31,45,46,49–58^, including both tissue and liquid biopsy samples from HCC, cirrhosis and healthy controls. We developed a machine learning pipeline to harness this resource to identify differentially methylated regions (DMRs) predictive of HCC in both tissue and liquid biopsies, from a background of cirrhotic samples. Our approach benchmarked favourably against 12 independent HCC methylation signatures and supported the development of a novel signature comprising 38 DMRs. Some of the identified regions were associated with transcriptional repression of several members of the Zinc Finger Proteins (ZFNs) family suggesting a potential role with cancer progression and early onset. Lastly, we combined the information of the novel DMR signature into a single score which successfully identified HCC tissue samples in an independent dataset (recall 96% and precision 98%), perfectly classified 13 healthy cfDNA samples, and identified 7 (out of 11) tumour cfDNA samples. Of note, the DMR signature score successfully identified cfDNA from diverse tumours, including colorectal and breast cancer, showing its potential as a diagnostic tool for multiple cancers. Overall, we present a systematic discovery and benchmark of methylation biomarkers for the early detection and monitoring of HCC using tissue and liquid biopsies and propose an improved signature and risk score with the potential to be used for non-invasive clinical diagnostics.

## Results

### DNA methylation dataset for the discovery of HCC biomarkers

**Figure 1:**
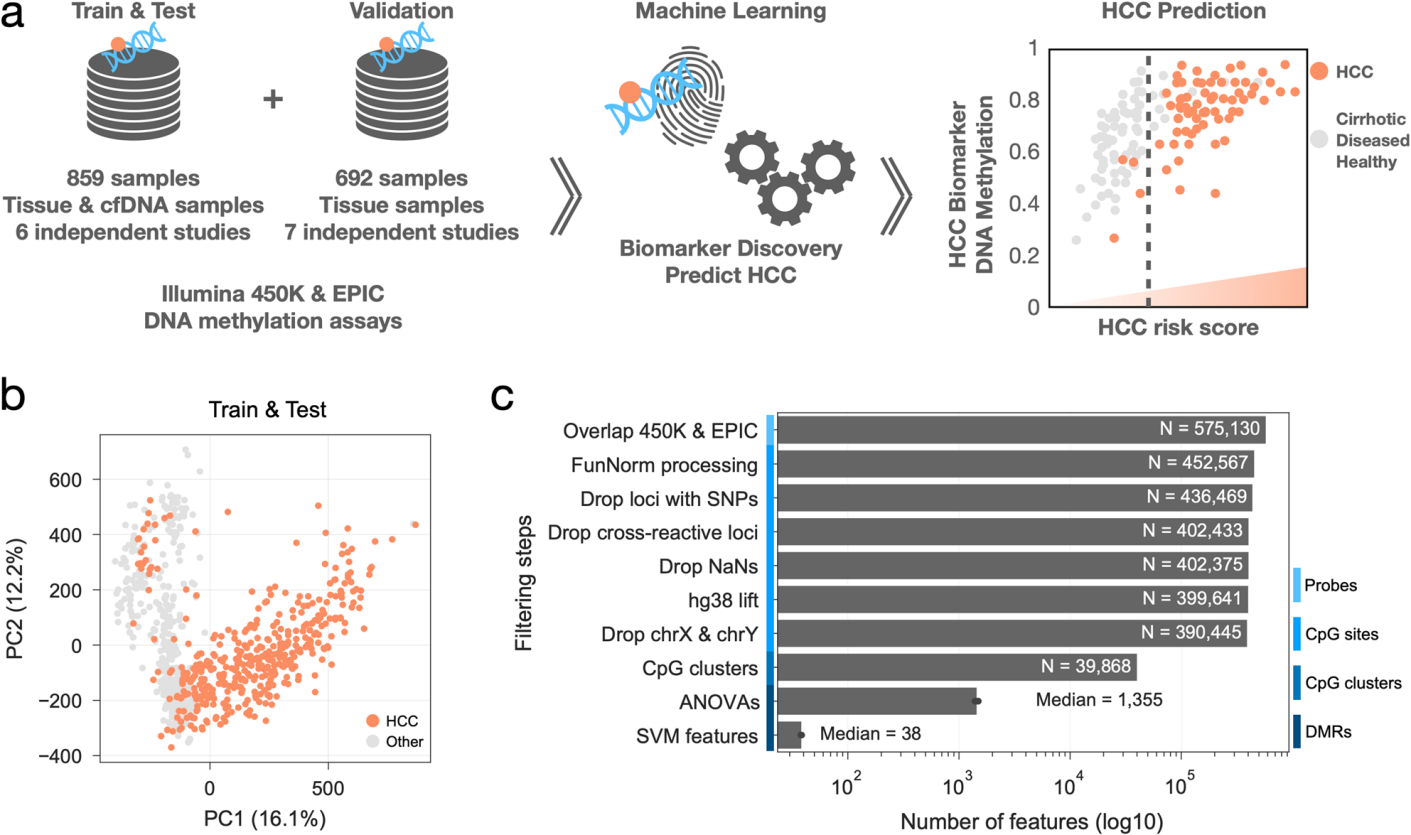
Data and workflow overview. (**a**) diagram depicting the different datasets assembled to discover Hepatocellular Carcinoma (HCC) DNA methylation biomarkers using machine learning approaches and to construct a HCC risk score, illustrative example. (**b**) principal component analysis (PCA) of the Train & Test DNA methylation dataset highlighting HCC samples. Principal component explained variances is shown within brackets. (**c**) Feature, i.e. probes, CpG sites, CpG clusters and differential methylated regions (DMRs), reduction steps across different stages in the processing and feature discovery pipeline.

To systematically discover DNA methylation biomarkers for the detection of HCC from tissue and plasma cfDNA samples we performed a comprehensive search of HCC-related studies and datasets characterising genome-wide DNA methylation changes (Fig. 1a). We queried commonly used data repositories, GEO^59,60^ and ArrayExpress^61^, using the keywords Hepatocellular Carcinoma, cfDNA and ctDNA. To ensure an exhaustive analysis of methylation markers we focused on studies that provided high-throughput assays and specifically Illumina-based, Infinium 450 K and EPIC assays, as these have been broadly adopted by large-scale studies. Additionally, to minimise potential undesired and technical batch effects while integrating multiple data sources for model training, only studies that provided raw unprocessed files were considered to allow the same processing pipeline to be applied to all samples^62–64^. Matching the criteria defined above we assembled 859 samples from 6 different studies^31,45,46,56–58^ covering: HCC and cirrhotic samples from tissue and cfDNA, including cirrhotic tissue from multiple aetiologies; healthy controls from both liver tissue and cfDNA; other non-HCC diseased tissue (e.g. liver obesity and Alpha 1 antitrypsin deficiency); and cfDNA from non-HCC patients (e.g. sepsis and other cancer types) (Fig. 1a,b and Supplementary Fig. 1a,b). A total of 452,567 methylation sites (CpG sites) are measured and methylation levels represented using beta methylation values, ranging between 0, unmethylated, and 1, fully-methylated. Additionally, we compiled a Validation dataset containing 692 tissue samples from 7 independent datasets^1,49–55^ for which original data or publication was not accessible but processed beta methylation values was available (Fig. 1a, Supplementary Fig. 1c). This validation dataset comprises multiple studies with distinct experimental and analytical pipelines and is intended to be used as independent validation of the approaches adopted in this study. Principal Component Analysis (PCA) reveals minimal grouping of the samples by dataset in the Train & Test dataset, while in the Validation dataset this is a bit more pronounced as it would be expected considering the samples from this dataset were not processed in a standardised way as the Train and Test dataset (Supplementary Fig. 1d,e). Most importantly, we observed that HCC or Other samples tend to cluster together even when their source dataset is different, i.e. TCGA and GSE60753, particularly in the Train and Test dataset which is the one used to train our models. Overall, we assembled 1551 whole-genome DNA methylation samples (Supplementary Table 1) representing an heterogenous and comprehensive resource to discover and benchmark DNA methylation biomarkers of HCC (Supplementary Fig. 1d,e) from clinically relevant diseased backgrounds, such as cirrhosis.

### Selection of high quality and informative DNA methylation regions

The assembled dataset measures > 450,000 CpG sites which is several orders of magnitude greater than the number of samples, thus posing a number of problems for training informative models. To mitigate this and to focus on high quality and informative measurements we applied several filtering steps to reduce the number of CpG sites, removing 14% of all features leaving a total of 390,445 CpG sites (Fig. 1c). Secondly, while a single CpG site can be informative and have strong predictive power of HCC status, due to the much larger number of CpG sites compared to the number of samples this can lead to spurious associations that are unlikely to be functionally relevant and generalisable to other cohorts, i.e. overfit. Considering that HCC patient samples showed distinct patterns of multiple and clustered CpG sites with hypo and hyper methylation profiles^58^, we searched for CpG clusters, spanning at least 3 CpG sites, such that two consecutive sites are at most 500 base-pairs (bp) apart. This defined a total of 39,868 CpG clusters with a median size of 700 bp spanning all 22 autosomal chromosomes (Fig. 1c, Supplementary Fig. 2a). For each CpG cluster we took the mean methylation of all CpG sites contained in it. Taken together, we performed an unsupervised reduction of the number of features by excluding problematic CpG sites and to focus on genomic regions, instead of individual CpG sites, to reduce the impact of potential confounder effects and help discover more generalisable biomarkers of HCC.

### Discovery of methylation regions predictive of HCC

**Figure 2:**
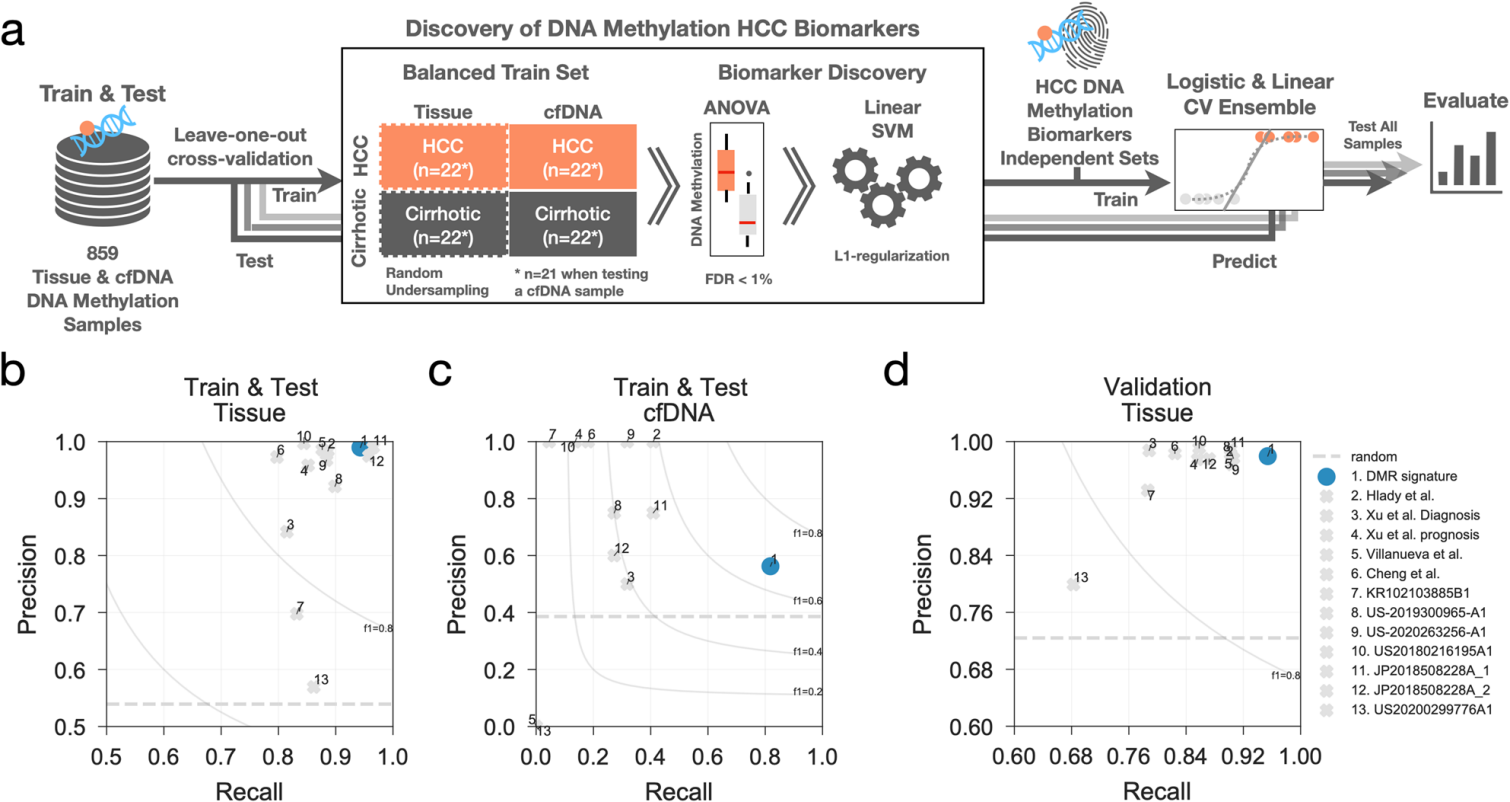
HCC biomarker discovery and benchmark pipeline. (**a**) machine learning workflow to identify DNA methylation regions predictive of HCC samples using balanced training sets and support vector machines and then benchmark against other independent DNA methylation biomarkers using an ensemble of logistic and linear regression classifiers. (**b**) precision and recall rates calculated over the leave-one-out test samples predicted using the logistic and ridge regression classifier ensemble. Similarly, precision and recall rates are calculated using the same ensemble but trained with CpG sites from independent HCC DNA methylation biomarkers and are compared. Here, only tissue samples of the Train & Test dataset are considered for the calculation of the precision and recall metrics. (**c**) similar to (**b**), instead precision and recall for cfDNA samples only are reported. (**d**) precision and recall rates obtained predicting the independent Validation set using the same ensemble trained with the multiple HCC biomarker feature sets measured in the Train & Test dataset.

To identify HCC from a background of cirrhotic samples in tissue and cfDNA we set out to find methylation regions predictive of HCC by training linear support vector machine classifiers (LinearSVC) (Fig. 2a). We applied a leave-one-out cross-validation strategy, where one sample at a time was left out for testing the prediction, while the other 858 samples were used as a training set. Considering there are many more tissue samples compared to cfDNA, this can create potential biases when training the LinearSVC (e.g. classes with more samples will weigh more on the importance of the features). To address this we balanced the number of samples of each class by randomly under-sampling the tissue samples to obtain 22 HCC (HCC-T) and 22 cirrhosis (C-T) samples, complemented with 22 HCC cfDNA (HCC-CF) and 22 cirrhosis cfDNA samples (C-CF). One balanced dataset per leave-one-out fold is generated ensuring that the sample left out for testing is not considered.

Differentially methylated and predictive regions are discovered using the balanced datasets in a two-step approach. Firstly, differentially methylated regions (DMR) are identified by removing potential cofounder effects, i.e. sex, age, global methylation and tumour purity. Considering that sex and age were not available for all samples, we estimated them from the DNA methylation arrays^62,65,66^. Global changes in methylation affect large swaths of CpG sites and thereby these do not represent optimal candidates for biomarkers due to their lack of specificity (Supplementary Fig. 2b,c). Lastly, the varying tumour purity of TCGA samples, the biggest source of HCC tissue samples in our analysis, has been quantified and represents a technical limitation that can affect molecular measurements, including DNA methylation, and their interpretation^67^. Tumour purity estimation is only available for TCGA samples. We observed from the PCA analysis of Train & Test dataset that PC5 is significantly correlated with tumour purity (Pearson’s r = 0.6, *P*-value = 3.28e−37). Therefore we considered PC5 as a proxy of tumour purity impact in the DNA methylation measurements (Supplementary Fig. 2d,e). A differential methylation analysis between HCC (HCC-T and HCC-CF) and cirrhotic (C-T and C-CF) samples was performed taking the previous variables as covariates in the linear model in order to discount their potential impact. Only significantly differentially methylated CpG clusters (likelihood-ratio test FDR < 1%) were selected for model training, thus reducing the number of features to a median of 1355 DMRs, across all leave-one-out folds (Fig. 1c). Secondly, DMRs are then used to train LinearSVC models for each cross-validation fold using a L1-regularization parameter to further reduce the number of DMRs to find the top predictive biomarkers of HCC. A median of 38 DMRs were selected per model (Fig. 1c). Taken together, this identified 150 DMRs that are present in at least 5% (n = 43) of all trained models (Supplementary Table 2) and the frequency of the DMRs in the optimal LinearSVC across the leave-one-out cross-validation is positively associated with their absolute mean effect size (Spearman rho = 0.29 and *P*-value = 1.9e−41, Supplementary Fig. 2f.).

In conclusion, the feature selection and model training steps performed in each cross-validated train set avoids information leak between train and test sets, addresses the problem of having many more features than samples and identifies the most predictive DNA methylation biomarkers of HCC.

### Evaluation, comparison and assembly of HCC methylation signature

Next, we set to define a DNA methylation signature predictive of HCC and compare it against independently defined sets. We estimated the optimal number of DMRs to consider in the methylation signature by sequentially testing the addition of DMRs into the feature set and tested the increment in precision and recall of the LinearSVC models (Supplementary Fig. 3a). Recall and precision shows the steepest increase up to 10 DMRs, and from that point the test and validation datasets show small but consistent increments in performance. Together with the fact that frequency of each DMRs in the optimal models is positively correlated with its absolute mean effect size, we selected the top 38 most frequent DMRs in the leave-one-out cross-validation procedure (Supplementary Table 3). The selected DMRs encompass hyper and hypo methylation events in HCC that are largely consistent across both Train & Test and Validation datasets and unsupervised clustering separates most HCC from non-HCC samples (Supplementary Fig. 3b).

We then benchmarked our DNA methylation signature against other similar approaches, assembling from the literature 12 sets of CpG sites proposed in 4 publications^1,31,47,68^ and 7 patents^69–75^. Notably, the DNA methylation sets were largely non-overlapping (Supplementary Fig. 4a) suggesting a disparity among HCC biomarkers and possibility indicating datasets-specific features which might not generalise well to other patient cohorts. To avoid potential bias to a specific method and to obtain better predictive performance we used an ensemble of logistic and linear classification models (Fig. 2a) (see Methods). For each leave-one-out cross-validation, the ensemble model was trained and used to predict the HCC status of the sample left out for testing. The performance of all models was estimated using multiple metrics, i.e. recall, precision, accuracy, Mathew’s correlation coefficient (MCC) and balanced accuracy (Supplementary Fig. 4b,c,d). It is important to note that most of the feature sets were derived using part of the DNA methylation datasets also utilised in this study, thus a complete independent validation of these feature sets was not possible, and it is expected that metrics will be overestimated. Overall precision and recall scores across the tissue are greater than 80% (Fig. 2b) and all models had a poorer performance when predicting the subset of cfDNA samples, while precisions were less affected (Fig. 2b,c). Our results were also robust to different cross-validation modalities, showing very similar precision and recalls using a fivefold cross-validator (Supplementary Fig. 4e) and high similarity indices between predicted labels (Jaccard similarity coefficient 0.942–0.997). We then used the Validation tissue samples dataset as an independent benchmark, and observed that overall feature sets provided a mean precision of 96% and recall rates of 86% (Fig. 2d and Supplementary Fig. 5), where our signature obtained the highest recall (95%) while preserving precision (98%) (Fig. 2d).

Collectively, our approach identifies a signature of hyper and hypo methylated regions that successfully distinguishes HCC samples from cirrhotic, healthy and other non-HCC samples, and benchmarks positively against other DNA methylation signatures, particularly showing low false negative rates, i.e. high recall, both in tissue and cfDNA samples.

### Molecular characterisation of methylation biomarkers

**Figure 3:**
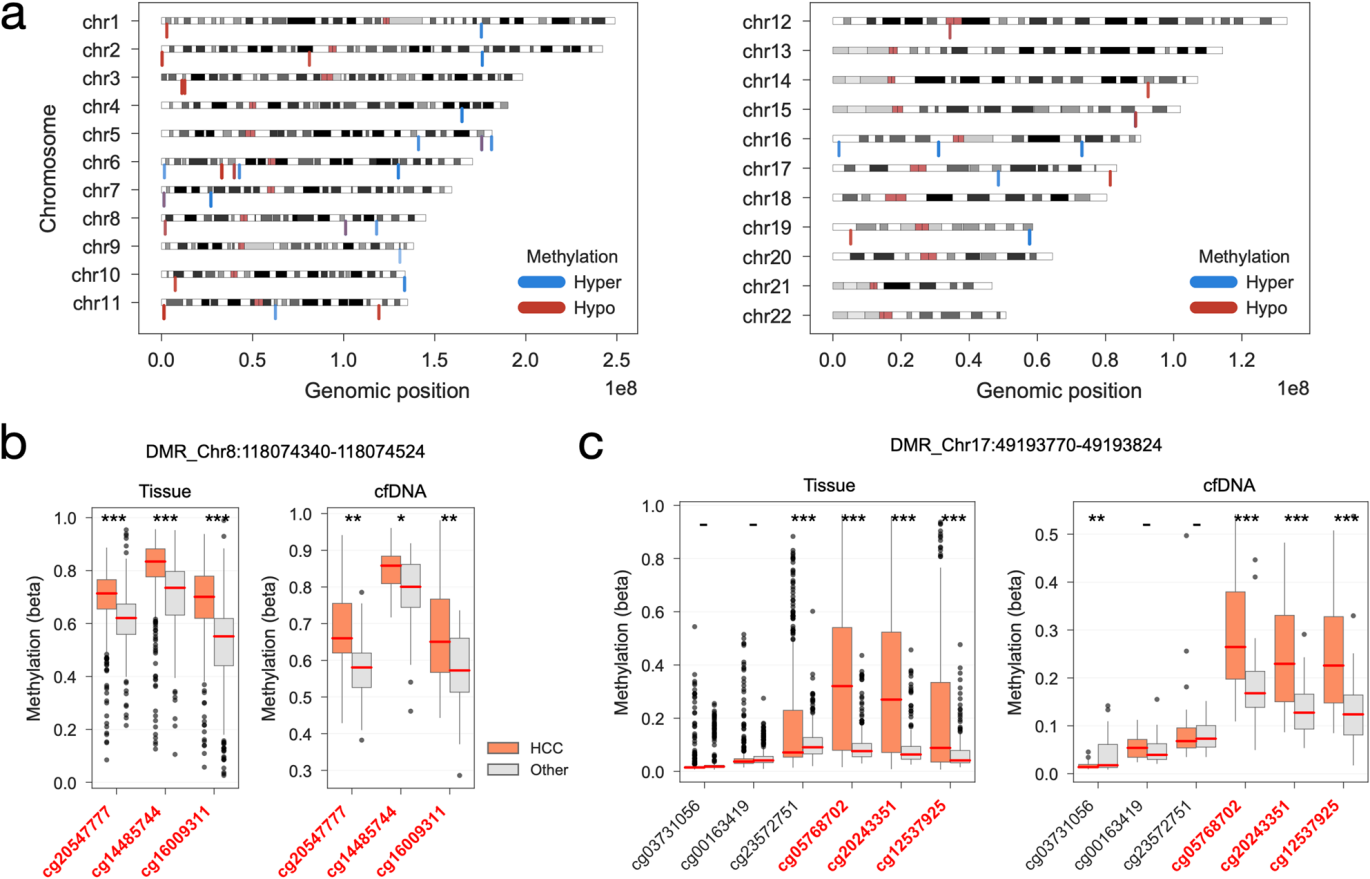
HCC DNA methylation biomarkers. (**a**) genomic localisation of the significantly differentially methylated CpG sites contained in the top 38 DMRs. Blue represents hypermethylation and red hypomethylation in HCC. (**b**) Top recurrent DMR in the optimal LinearSVC models. Distribution of DNA methylation (beta) of CpG sites contained within 1000 base-pairs up/down-stream of the DMR. In red are labeled CpG sites that are contained in the DMR. DNA methylation is split and coloured by HCC and the rest. Left panel shows the methylation of all tissue samples in the Train & Test dataset, and right-hand side the DNA methylation of cfDNA HCC, cirrhotic and healthy samples. Above the plots are reported the DMR associated chromosome and genomic coordinates. (**c**) similar to (**b**), instead showing the distribution of a representative DMR that is highly predictive of HCC in both tissue and cfDNA samples. *, ** and *** denotes significant at *P* < 0.05, *P* < 0.01 and *P* < 0.001 by unpaired t-test, respectively, and ‘-’ otherwise.

Having assembled a methylation signature of HCC, we then set out to molecular characterise it in more detail. The top 38 DMRs encompasses a total of 214 CpG sites out of which 118 and 74 showed significant hyper and hypo methylation in HCC, respectively (Fig. 3a, Supplementary Table 3). Reassuringly, inspecting the top DMRs showed that the methylation of the CpG sites within each cluster is able to clearly separate between HCC and non-HCC samples in both tissue and cfDNA samples (Fig. 3b,c). We further explored this by taking advantage of the availability of gene-expression datasets for 410 liver samples from the TCGA consortium^56,76^, and systematically tested associations between the 38 DMRs and 15,341 gene expression profiles. We identified a total of 39 significant DMR-gene associations (linear regression log-likelihood ratio test FDR < 10%, Supplementary Table 4). Among the top associations are several positive associations between DMR Chr7:27,144,326–27,145,664 and multiple members of the homeobox transcription factors (HOXA6, HOXA3, HOXA5, HOXA7 and HOXA4) (Supplementary Fig. 6a) which are all close to the DMR and have been suggested to be involved in tumorigenesis and cell proliferation and migration^77,78^. While positive associations, i.e. increase in methylation associated with increased gene expression, might be related with potentially more complex regulatory mechanisms, negative associations might capture decreased gene expression through repression of transcription due to hypermethylation. We observed multiple negative associations with Zinc Finger Proteins (ZNF518B, ZNF502 and ZNF132) (Supplementary Fig. 6b). The role of the Zinc Finger Proteins in cell adhesion and in cancer is well described^79,80^ and could highlight some of the biological mechanisms underlying hypermethylation of these regions in HCC (Supplementary Fig. 6c). In summary, the methylated DNA regions highlighted with our approach, are potential useful biomarkers for HCC and may also reveal important biological information, specifically ZNF518B and its associated DMR, Chr10:133,445,694–133,446,718, is among the most important features and has been previously described with possible implications in cancer cell invasion and metastatic potential^81^.

### Diagnostic score based on HCC methylation signature

**Figure 4:**
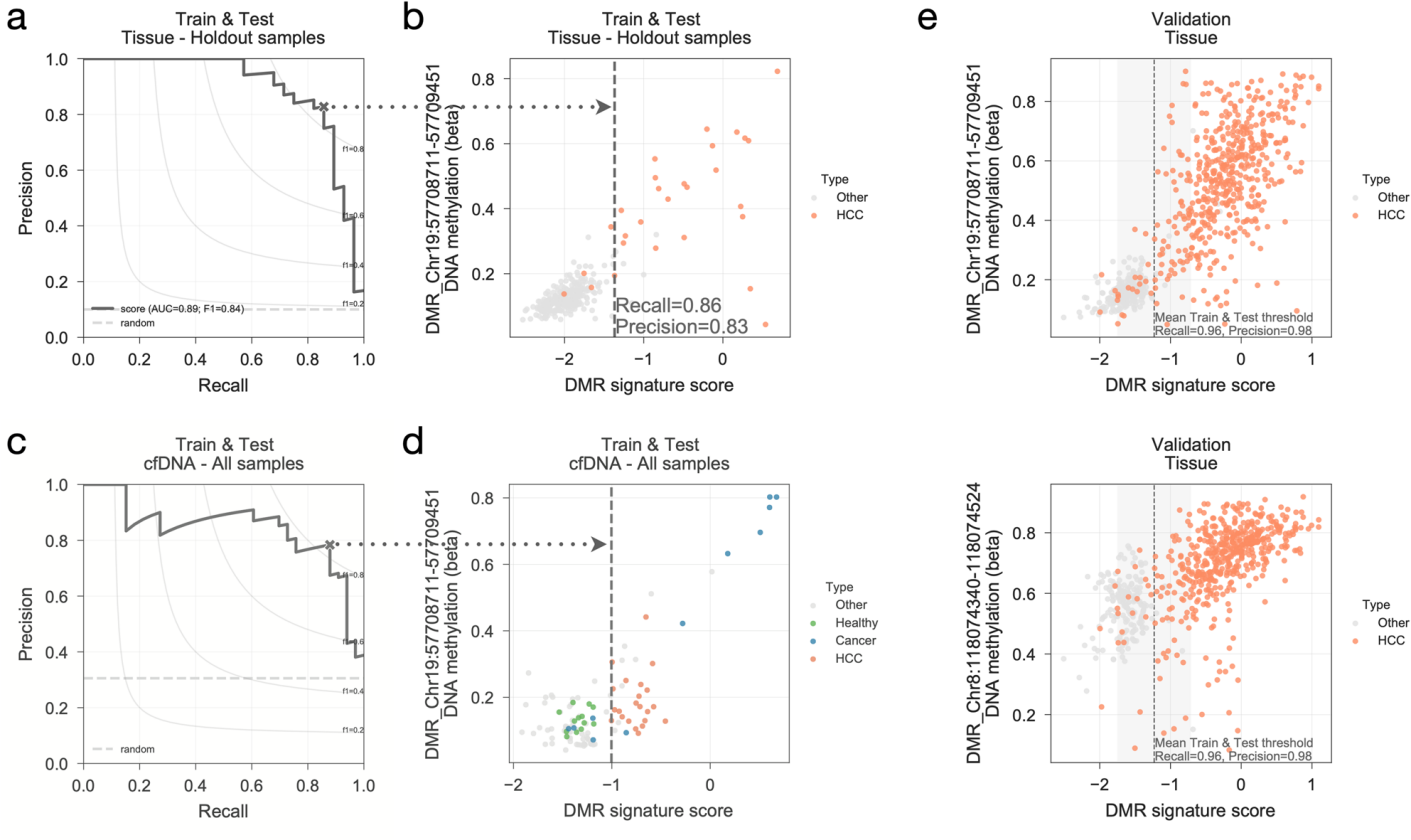
HCC DMR signature score. (**a**) precision-recall curve using the DMR signature score ranking the samples in the Train & Test dataset that were not used to define the DMR signature, i.e. were left out when training the models to identify the 38 DMRs and their associated weights. Maximum F1-score along the curve is represented with “x” and used to define the DMR signature score threshold at the given recall and precision. Random precision is drawn as a dashed horizontal line. (**b**) DMR signature score of Train & Test samples not used for HCC biomarker discovery plotted against a representative top performing DMR. Vertical line represents the DMR signature score threshold found at the maximum F1-score in (**a**) and the associated recall and precision rates are reported. (**c**) precision-recall curve of all cfDNA samples of the Train & Test dataset including samples from patients with other types of cancer (labeled as “Cancer” and coloured blue). (**d**) similar to (**b**), DMR signature score threshold, vertical dashed line, is estimated from the maximum F1-score point along the precision-recall curve in (**c**). (**e**) DMR signature score calculated for the Validation set samples plotted against two highly predictive HCC DMRs and their methylation profiles. Precision and recall rates reported are those estimated in the Validation dataset using the DMR signature score threshold calculated with the Train & Test dataset.

Lastly, we defined a single metric that could encompass the information from a whole DNA methylation signature to use as a diagnostic metric for early detection of HCC. First, we robustly estimated the coefficients of each DMR in the signature by randomly generating 1000 balanced training datasets, as described before (Fig. 2a), and training a regularised linear regression classifier (Supplementary Fig. 7a). Sorted in descending order of their absolute coefficients, the top 8–10 DMRs in the signature contribute most to the recall of HCC in the Validation dataset using the Train and Test dataset for training, while the remaining DMRs provide smaller but consistent improvement (Supplementary Fig. 7b). Secondly, we built an additive linear score (DMR signature score) where each 38 DMRs of the methylation signature is weighted by their signed mean coefficients, i.e. DMRs with high absolute mean coefficients have higher preponderance in the score. For all samples in the Test and Train and Validation dataset we calculated their DMR signature score and ranked them into how probable they are from being HCC (Supplementary Table 5). Similarly, we estimated a linear risk score for the other CpG site signatures, and observed that in the independent Validation dataset the score based on our DMRs signature outperformed and provided very accurate predictions of HCC (Supplementary Fig. 7c). Furthermore, in samples from the Train & Test dataset that were held out from the training of the DMR signature and score could achieve a clear split between the HCC compared to non-HCC samples with a recall (sensitivity) of 86% and precision of 83% (Fig. 4a,b).

We also looked in particular to the cfDNA samples which have noisier backgrounds in terms of methylation signals and are more relevant for non-invasive early-stage diagnostic approaches based on blood liquid biopsies. In addition to the HCC and cirrhotic cfDNA samples, we also considered cfDNA samples of healthy controls, sepsis and patients with cancers from other tissues, including lung, breast and colon^57^. Not surprisingly our metric could separate cfDNA HCC and cirrhotic samples, which are used for training of the signature and score. More interestingly, it perfectly splitted independent healthy control samples and could identify cfDNA samples from patients with other cancers (Fig. 4c,d), supporting the capacity of our signature and associated score.

Altogether, the linear risk score represents a metric for the diagnosis of HCC that showed robust predictive power across many different datasets (Fig. 4e) with heterogeneous backgrounds and most importantly both in tissue and liquid biopsies (Supplementary Fig. 7d,e). While the recall and precision metrics reported here are limited to the amount of cfDNA datasets available these results suggest that DNA methylation from plasma cfDNA is a promising alternative to AFP-based approaches.

## Discussion

Hepatocellular carcinoma (HCC) diagnosis is challenging and often misses early detection which is vital to ensure curative options are available to the patient. Non-invasive diagnostic approaches based on serum biomarkers, such as alpha-fetoprotein (AFP), AFP isoforms and micro-RNAs, have shown sub-optimal sensitivity, leaving many patients undiagnosed. Tumour cell-free DNA (cfDNA) from blood liquid biopsies holds great promise to transform clinical oncology diagnosis^26,43,82,83^ with several studies reporting highly specific methylation signatures for the diagnosis and prognosis of HCC^1,31,47,68^. Currently, most HCC methylation signatures are small sets of single CpG sites (median n = 7) and overall show poor agreement between them. This might indicate these signatures are potentially specific to the studies, which could hinder generalisation to other cohorts and the utility for liquid biopsies as these are noisier backgrounds with low available materials, thus affecting the detection of these very specific features. To address this, we assembled > 1500 genome-wide DNA methylation arrays from 13 independent datasets^1,31,45,46,49–58^ making this one of the largest methylation compendium to study HCC to date. We harnessed this rich dataset by implementing a machine learning pipeline that searches, in an unbiased way, for significantly differentially methylated regions (DMRs) in HCC presenting several improvements. Firstly, considering regions spanning multiple CpG sites increases confidence as these can be more robustly measured in liquid biopsies in clinical settings. This procedure reduces the impact of eventual CpG site misdetection in the diagnosis and makes this more amenable for next-generation sequencing readouts, which measure all sites within the specified region. Secondly, training machine learning predictors with a training set equally representing tissue and liquid biopsies ensures the DMRs identified are representative of HCC tumours that can also be measured in ctDNA. Moreover, making this comparison against a cirrhotic background, instead of healthy liver samples, provides a more relevant clinical comparison. Very often patients who develop HCC also suffer from chronic liver disease and cirrhosis, and these are the backgrounds where existing non-invasive alternatives underperform. Lastly, to reduce potential analytical artefacts in the DMR biomarker discovery we processed the training dataset (859 samples from 6 different studies^31,45,46,56–58^) from raw data with the same pipeline and applied stringent filters to remove problematic measurements and account for potential confounders, such as sex, age, tumour purity and global methylation, often not considered by other studies. Additionally, we validated our approach using not only hold-out samples and cross-validated procedures, but also an assembled validation dataset (692 samples from 7 independent datasets^1,49–55^), which was never used for training and comprises differently and independently processed datasets, thus testing the robustness of our DMRs to diverse processing pipelines.

Our machine learning approach compared favourably against 12 HCC methylation signatures^1,31,47,68–75^ across multiple datasets in both tissue and liquid biopsy samples. We harnessed this to derive a novel methylation fingerprint comprising 38 DMRs and combined it into a single diagnostic metric which detected HCC tissue samples in a validation dataset with 96% recall and 98% precision.

A limitation of our analysis is linked with the scarcity of cfDNA methylation samples. While this is ubiquitous across other independent studies, it limits the estimation and extrapolation of evaluation metrics, recall and precision, to other cohorts. To mitigate this, we thoroughly benchmarked our approach by assembling comprehensive and independent training and validation DNA methylation datasets. Specifically, we aimed to integrate as many liquid biopsy samples as possible, e.g. cfDNA analyses from healthy controls, sepsis and different tumours^57^, and while not directly related with HCC these samples supported the utility of out approach, by for example showing it could correctly classify all healthy cfDNA samples. Of note, the DMR signature score also successfully identified 7 cfDNA samples (out of 11) from other tumours, including breast, lung and colorectal cancer.

This last point suggests that our ctDNA methylation signature and risk score have the potential for pan-cancer early diagnostics. Indeed other studies have shown that DNA methylation biomarkers can be used for the detection of different cancers, such as promoter methylation of the gene SEPT9 in colorectal cancer and HCC^26,43,57,84^. Gene expression analysis showed that several DMRs of our signature are significantly associated with transcriptional repression of multiple Zinc Finger Proteins (ZFNs) supporting a potential role of these regions in cancer progression and early onset^80,85^. Caution needs to be taken when interpreting this signature as a potential pan-cancer detection as it will be of limited use to identify the tissue of origin of the tumour. This is intended to be used in the context of HCC where, for example, cirrothic patients are clinically followed and this DNA methylation would support the decision for further diagnosis of HCC. Lastly, this approach is currently being considered to monitor HCC patients that have undergone therapies, such as surgical liver resection, radiofrequency ablation and chemoembolization, as a means of clinical follow-up to identify residual disease and guide treatment^12,86^.

In this study, we present a machine learning pipeline that harnesses a comprehensive genome-wide DNA methylation resource to build a signature and a diagnostic score for HCC that benchmarks favourably against existing biomarkers. While further work to confirm the clinical utility of this approach is ongoing, it addresses important challenges of the design of reliable non-invasive diagnostic and monitoring approaches for HCC from liquid biopsies, to provide long sought-after alternatives to current suboptimal approaches.

## Methods

### DNA methylation datasets assembly and processing

DNA methylation samples from 6 different datasets^31,45,46,56–58^ using Infinium HumanMethylation EPIC and 450 K assays were processed using the R package minfi (v1.32.0)^62,64^. Datasets were integrated by considering the overlapping CpG probes between the two Infinium HumanMethylation assays (n = 575,130). All datasets were merged into a single matrix containing signal intensities imported from the raw IDAT files and processed using the functional normalisation pipeline^63^. Lastly, the ratio between the methylation and unmethylated channels was calculated and exported as beta values (*β*) Eq. (1) with an offset of 100 and rounded to 5 decimal places:

1 $$ \beta = \frac{Methylated}{{Methylated + Unmethylated + offset}} $$

Altogether, we generated a single matrix of DNA methylation beta values spanning 452,567 CpG sites measured across 859 samples, integrating multiple studies processed from the raw signals using the same pipeline. For the downstream analyses several filtering steps were taken: (1) probes containing a single nucleotide polymorphism (SNP) in the CpG site or in the single nucleotide extension at a minor allele frequency (MAF) greater or equal than 0.01 were excluded from downstream analysis; (2) using maxprobes R package (v0.0.2, https://github.com/markgene/maxprobes) cross-reactive probes of the Illumina methylation arrays were removed^87–90^; (3) CpG sites with missing values were discarded; (4) we utilised an updated probe annotation mapped to the hg38 reference build and probes with no available alignments were not considered; and (5) to focus on biomarkers that are sex agnostic CpG sites mapping to sex chromosomes X and Y were removed from downstream analyses. The final filtered DNA methylation matrix covered a total of 390,445 CpG sites without any missing value across all samples.

### DNA methylation regions, CpG clusters

To identify DNA methylation regions, CpG clusters, we utilised a similar approach to the one described in Jaffe et al.^91^. Using the clusterMaker function from Bump Hunter R package (v1.30.0)^62,91^ we identified CpG clusters with a maximum of 500 base-pairs (bp) distance between any 2 consecutive CpG sites. Then we overlapped the CpG clusters with the filtered CpG sites defined previously and only considered CpG clusters with at least 3 CpG sites with measurements. A final CpG cluster matrix was defined by taking the mean of all filtered CpG sites within each cluster region, generating a DNA methylation matrix spanning 39,868 CpG clusters.

### Dimension reduction analyses

Dimension reduction analysis was performed using Principal Component Analysis (PCA) implemented in scikit-learn Python module (v0.24.0)^92^.

### Balancing training samples sets

Considering the number of samples in each class, i.e. HCC, cirrhotic, cfDNA and tissue, the Train and & Test is highly unbalanced and this can generate artefacts that can limit an unbiased discovery of HCC biomarkers (Supplementary Fig. 1a). Thus, we balanced the number of samples in each type for the training of the machine learning models. Since the limiting number of samples are from cfDNA samples, all samples available for HCC (n = 22) and cirrhotic (n = 22) from cfDNA are used for training. Then an equal number of samples (n = 22) for HCC and cirrhotic are randomly sampled from the tissue samples, specifically Primary Tumour—Liver for HCC class, and Cirrhosis + HBV, Cirrhosis + HCV, Cirrhosis + AATD and Cirrhosis + EtOH for the cirrhotic class. Some cirrhotic tissue samples from the same dataset showed very distinct profiles diverging from other cirrhotic samples, thus we excluded them from the generation of the balanced dataset by considering only those cirrhotic samples from the GSE60753 dataset^58^ with a Principal Component (PC) 2 lower than 200 (Supplementary Fig. 8a,b). Taken together, a total of 88 samples, evenly separated by HCC and cirrhotic and cfDNA and tissue, are used for model training (Fig. 2a). Within the leave-one-out cross-validation procedure, see below, in the cases where the test sample is a cfDNA sample this sample is not used for training and the total number of samples in each class is therefore reduced to 21, hence a total of 84 evenly distributed samples are used instead.

### Discovery of HCC biomarkers using support vector machine classifiers

The systematic search of DNA methylation biomarkers of HCC and benchmark against other independent sets of biomarkers^1,31,47,68–75^ was performed within a leave-one-out cross-validation procedure across the 859 samples contained in the Train & Test dataset. In this procedure one sample at a time is left out for testing and the rest are used to build a balanced dataset (undersampling of the HCC and cirrhotic tissue samples) to identify differentially methylated regions (DMRs) predictive of HCC.

Firstly, with the balanced train dataset we defined DMRs using a multivariate linear regression model, LinearRegression class from scikit-learn (v0.24.0), that takes as dependent variables the mean methylation values of the 39,868 CpG clusters contained in the balanced dataset (Samples x CpG clusters) and as independent variable (Samples × 1) the binary classification if a sample is HCC (1) or not (0). Additionally, multiple potential confounding factors, covariates, are included in the model as independent variables: (1) binary variable representing sex (female), since this information in incomplete, we accurately estimated the sample sex using the methylation profiles and the R package minfi (v1.32.0)^62,64^; (2) patient age, this is also largely unavailable and therefore we used^65,66^ the R package wateRmelon^93^ (v1.0.0) to estimate methylation age of the sample using their methylation profile and considered the Hannum^65^ and Horvath^66^ approaches; (3) sample global methylation, to mitigate potential biases mediated by the sample overall methylation levels we calculated the sample mean methylation levels and considered it as another independent variable; (iv) tumour purity, this information is only available for the TCGA samples^56,76^, CPE purity^67^, and the varying levels of tumour purity affect the molecular measurements and thereby we included Train & Test PC5 in the model as a proxy to tumour purity estimations (Spearman’s rho 0.59, *p*-value 9.6e−37); and lastly (v) we included an intercept term. The full model is fitted and a beta coefficient is estimated for each independent variable. To statistically assess those CpG clusters that are significantly differentially methylated in HCC we also trained a smaller model (null hypothesis) that excludes the HCC status to test the hypothesis that the CpG cluster methylation status provides a significant increase in the classification power of HCC over the covariates. This is estimated using the log-likelihood ratio test for every CpG cluster and the *p*-values are then adjusted for multiple-hypothesis testing using the Benjamini–Hochberg False Discovery Rate (FDR). We complement this with a ANOVA differential CpG cluster methylation analysis performed with the *f_classif* function from the scikit-learn (v0.24.0)^92^ module and statistical assessment using the F-scores associated p-values after adjusting for multiple hypothesis with FDR. Lastly, DMRs are defined as those CpG clusters with a ratio test and ANOVA FDR lower than 1%. This identified a median of 1,355 DMRs across the leave-one-out procedure.

Secondly, having identified DMRs in HCC we then estimate the most important DMRs to predict HCC by training linear support vector machines (LinearSVC) using a L1 regularization, with penalty parameter (C) set to 1.5, to reduce the number of DMRs considered in the model. DMRs with non-zero weights in the trained model are then defined as the most predictive DMRs to classify HCC samples. A median of 38 HCC predictive DMRs are identified per model across the 859 folds of the leave-one-out procedure, where 150 unique DMRs are found in at least 5% of all trained models (n = 43).

### Benchmarking DMRs against other DNA methylation signatures

For each leave-one-out cross-validation, the top predictive DMRs identified in the Biomarker Discovery section (Fig. 2a) (DMRs with non-zero coefficients in the LinearSVC model) are used to train an ensemble of Logistic and Ridge linear classifiers. For each method an internal cross-validation is performed to estimate the regularisation parameters C and alpha, respectively. HCC binary classes are calculated using the VotingClassifier class from scikit-learn (v0.24.0)^92^ which uses a soft voting modality, i.e. taking the argmax of the estimated probabilities to be HCC. For training the ensemble all samples from the Train & Test dataset are used, apart from the one left out for testing. In contrast to the DMR discovery, training of the ensemble is not restricted to the balanced sample set. Lastly, the trained ensemble model is used to make a prediction of the HCC status of the test sample using the soft-voting.

A similar procedure is performed for the 12 independent HCC DNA methylation signatures, training an ensemble model per signature restricted to the CpG sites contained in the signature, and then a prediction is made about the HCC status of the test sample. All predictions on the test sample are stored and multiple evaluation metrics are calculated compared to the true label: confusion matrices, recall, precision, sensitivity, balanced accuracy, and Mathew correlation coefficients (MCC).

### Sequential feature selection

The forward greedy sequential feature selection procedure shown in Supplementary Fig. 3a iteratively finds the optimal number of DMRs using the Train & Test dataset, i.e. assesses when the predictive power to classify HCC plateaus compared to the number of DMRs used. This was performed using the SequentialFeatureSelector function implemented in the python module scikit-learn (v0.24.0)^92^. For this analysis only DMRs that were found in more than 5% of the leave-one-out optimal models (n = 43 models) were considered (n = 150 DMRs). Considering that 38 is the median optimal number of features in the leave-one-out cross-validated models (Fig. 1c) and that the frequency of the DMRs is positively correlated with its absolute effect size (Supplementary Fig. 2d), we set this as the maximum number of DMRs to consider. We trained and tested LinearSVC models with a ranging number of DMRs from 1 to 38, for each model we utilised a balanced dataset for training and repeated this 30 times for each number of DMRs. In each model, predictions for the train, test and validation samples were performed and evaluated with precision and recall metrics (Supplementary Fig. 3a).

### Linear regression models between gene expression and methylation

To identify potential associations between DMRs methylation and gene expression we utilised transcriptomics measurements^56,76^ available for the liver TCGA samples contained in the Train & Test dataset (n = 410). Within this subset we systematically tested linear associations between methylation profiles of the top 38 DMRs and 15,341 gene expression profiles using linear regression models implemented in the python module limix (v3.0.4)^94^. We defined the following linear mixed model Eq. (2):

2 $$ {\text{y}}_{{\text{m}}} = {\text{ b}}_{{1}} {\text{M }} + {\text{ b}}_{{2}} {\text{X}}_{{\text{e}}} + {\text{ b}}_{{3}} {\text{K }} + {\text{ e}} $$

where *y* represents a vector of a single DMR methylation profile, *M* represents a matrix of covariates, *x* a gene expression vector of a single gene, *K* is the random effects term represented by the Kinship matrix of all the samples estimated using a linear kernel, and *e* is the noise term. The covariate matrix, *M*, contains several factors that might confound associations, similar to before: (1) global methylation; (2) predicted patient sex; (3) predicted patient age using both Hannum and Horvath methods; (4) tumour purity, Train & Test PC5 used as a proxy; and (5) an intercept term. Gene expression measurements were standardized by subtracting the mean and dividing by the standard deviation. For each DMR and gene association a Eq. (2) linear mixed model was fitted by minimising the residual sum of squares to estimate the parameters $b_{1}$, $b_{2}$ and $b_{3}$. Statistical significance was assessed by performing a log likelihood ratio test between the full model Eq. (2) and the null model which excludes the gene expression term ($b_{2} x_{e}$), *p*-value was derived using a chi-square distribution with one degree of freedom and correction for multiple testing using FDR. A total of 582,958 DMR and gene expression associations were tested and 39 were found to be significant at a FDR < 10%.

### HCC DMR signature score

HCC linear risk score (DMR signature score) is a sample score estimated from our 38 DMR signature. This is calculated using a weighted sum of the methylation of the 38 DMRs recurrently present with non-zero weights in the linear support vector machines (LinearSVCs) trained with the balanced sample sets in the leave-one-out cross-validation. The preponderance (weight) of each DMR is independently estimated using 1000 permutations of the balanced datasets which are used to train a Ridge classifier with an alpha parameter set to 1. This ensures a regularisation of the model’s feature coefficients, while preserving them to non-zero. The mean and standard deviation of each DMR is then calculated across all 1000 iterations. The mean coefficients are used to weight the DMR signature score, where features with larger absolute coefficients have larger preponderance. A score is calculated for each sample using the sample-specific DMR methylation values and the weights calculated before. Recall and precision curves are generated using the risk score and the HCC status of the samples. Optimal threshold and precision and recall rates are estimated based on the best F1 metric possible along the curves. A similar approach is taken for the other 12 independent DNA methylation signatures, where CpG sites are used as features instead.

## Supplementary Information

Supplementary Information 1.

Supplementary Information 2.

Supplementary Information 3.

Supplementary Information 4.

Supplementary Information 5.

Supplementary Information 6.

## Data Availability

The datasets analysed during the current study are publicly available in the referred studies. Data and source code used to perform the analyses described in this study are provided as supplementary materials.
