# Supplementary material for: DNA methylation fingerprint of hepatocellular carcinoma from tissue and liquid biopsies
Source: Sci Rep. 2022 Jul 7;12:11512. doi: 10.1038/s41598-022-15058-0 (PMC9262906; doi:10.1038/s41598-022-15058-0)
Supplement: Supplementary file 1 — Supplementary Information 1. [file 41598_2022_15058_MOESM1_ESM.docx]

# **DNA methylation fingerprint of hepatocellular carcinoma from tissue and liquid biopsies**

Emanuel Gonçalves, Maria Gonçalves-Reis, José B Pereira-Leal, Joana Cardoso  ^#^

Ophiomics, Pólo Tecnológico de 8, R. Cupertino de Miranda 9, 1600-513 Lisboa, Portugal

# Corresponding author. Email: [jvaz@ophiomics.com](mailto:jvaz@ophiomics.com)

Keywords: HCC; DNA Methylation; Liquid Biopsies; Early Detection; Monitoring

##

## **Supplementary Figures**


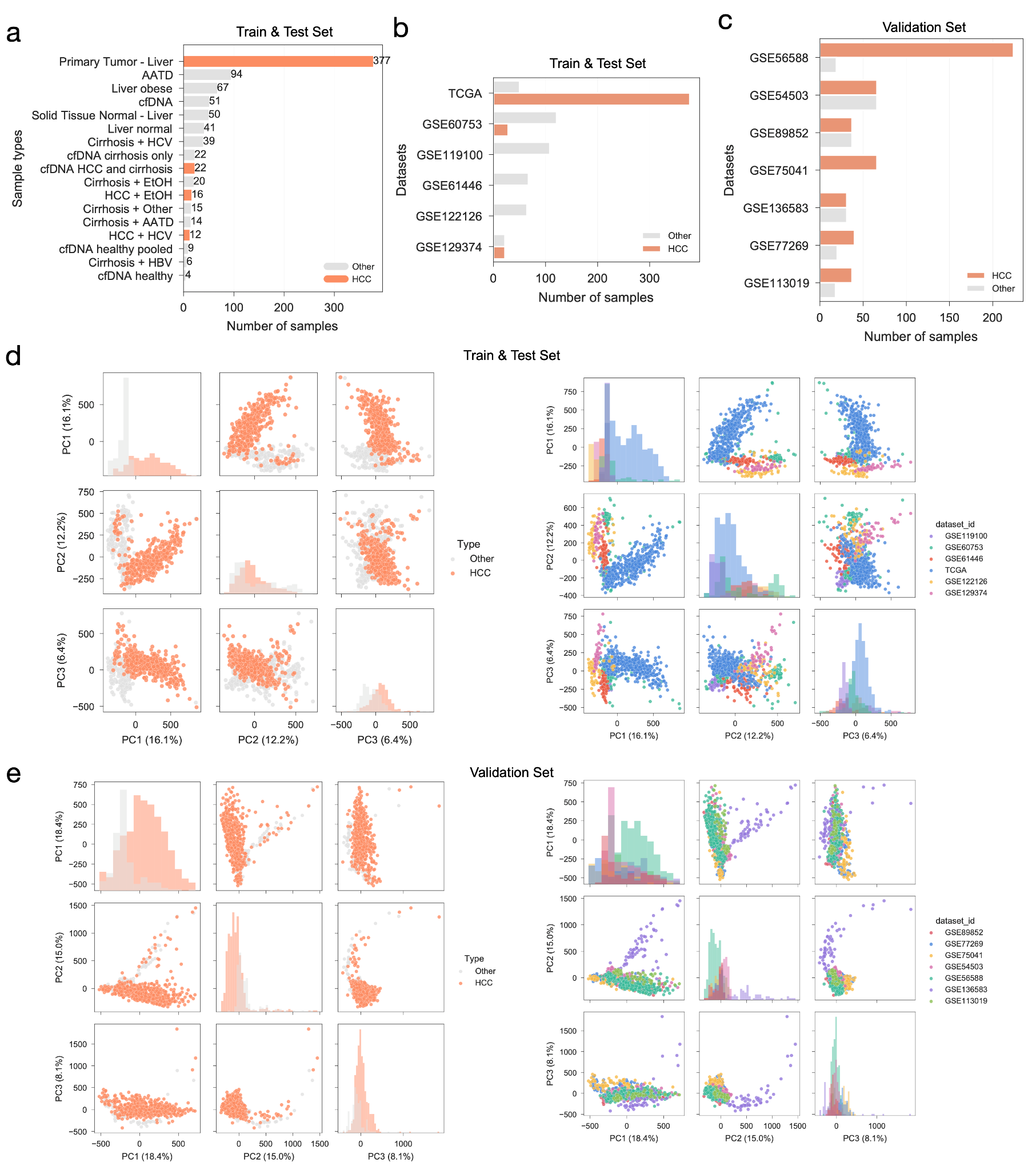


**Supplementary Figure 1. Overview of the DNA methylation datasets assembled.** a) number of samples across different types, i.e. HCC, healthy, cirrhotic and other diseased liver samples. b) number of samples per study constituting the Train & Test dataset. c) similar to b), number of samples per study constituting the Validation dataset. d) principal component analysis (PCA) of the Train & Test dataset, plotting the first 3 principal components against each other (off-diagonal) and the distribution (diagonal). HCC samples are highlighted from the rest (left panel), and samples coloured by dataset (right panel). e) similar to d), PCA of the Validation dataset.


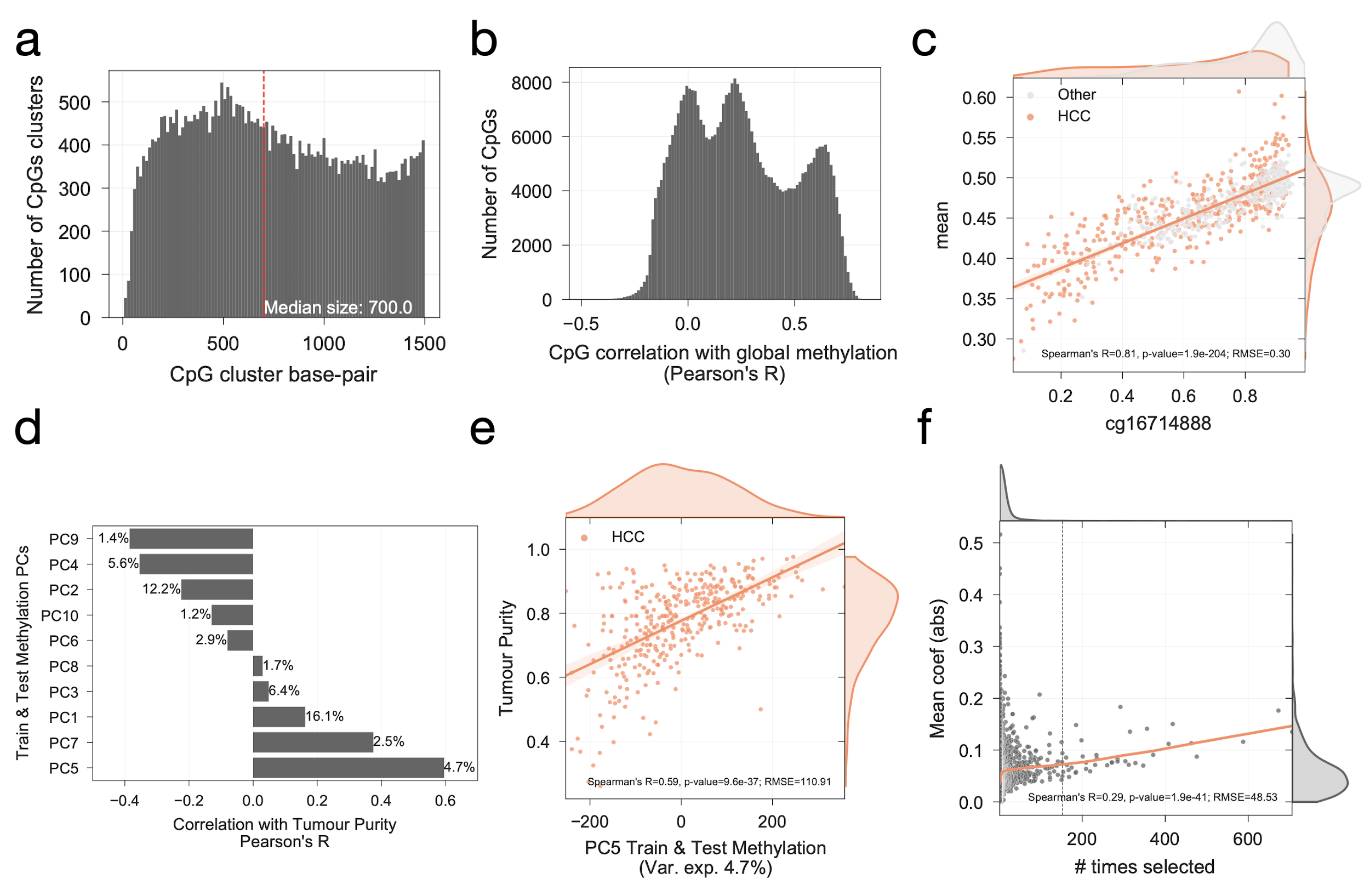


**Supplementary Figure 2. Information of DNA methylation features.** a) distribution of the length of the CpG clusters. Median size of all clusters is represented as a vertical line. b) distribution of Pearson’s correlation coefficient (R) between all CpG sites contained in the Train & Test dataset and the overall sample mean methylation. c) top correlated CpG site with mean DNA methylation. HCC samples from the Train & Test dataset are highlighted in orange from the rest. d) Pearson’s correlation coefficients between the first 10 principal components (PCs) of the Train & Test methylation dataset with estimated tumour purity [^67^](https://paperpile.com/c/dPipfM/HKMT) for the HCC samples from the TCGA dataset [^56^](https://paperpile.com/c/dPipfM/aJit0). PCs explained variance is reported next to the corresponding bar. e) Scatter and linear regression of top correlated Train & Test methylation PC, PC5, with TCGA HCC samples estimated tumour purity. f) number of times a DMR is present in the optimal LinearSVC model in the leave-one-out cross-validation procedure plotted against the mean absolute coefficient. Dashed vertical line represents the frequency cut-off of the top 38 DMRs.


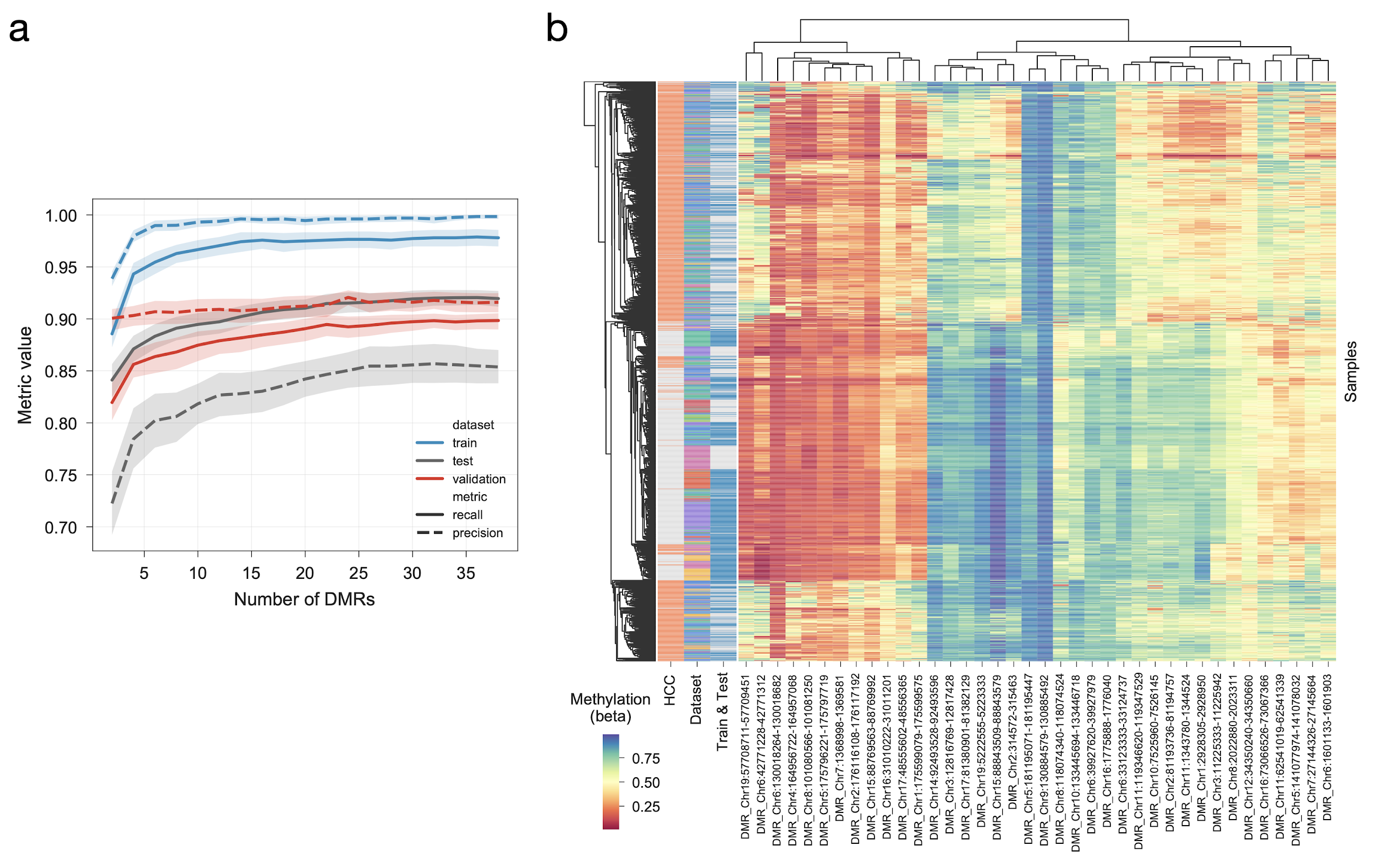


**Supplementary Figure 3. Top DNA methylation HCC biomarkers.** a) greedy sequential DMR selection of the best DMR is selected to be added to the LinearSVC model. For each number of DMRs, 30 balanced train sets were generated and benchmarked. Models were trained with balanced train sets and used to predict the train, the test and the validation datasets. The number of features to be selected ranges from 1 to 38, where the latter represents the median number of features in the LinearSVC models. Error margins represent the 95th confidence interval. b) DNA methylation heatmap of top 38 DMRs across Train & Test and Validation datasets. Row colour annotations identify, in order: HCC samples (red) from the rest (gray); the different datasets used; and the samples that correspond to the Train & Test dataset (blue) from the Validation dataset (gray).


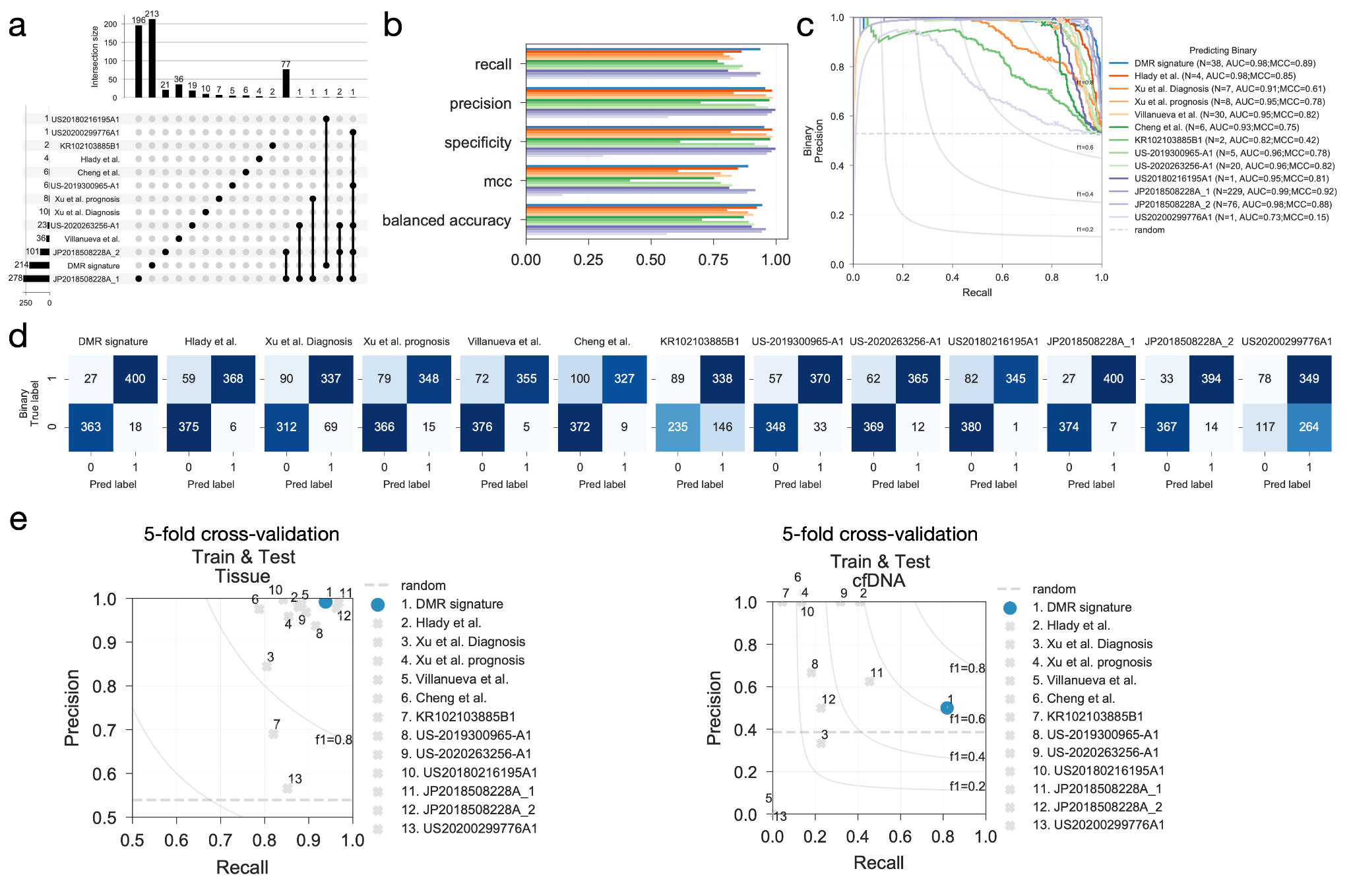


**Supplementary Figure 4. Train & Test dataset trained models performance.** a) UpSet plot showing the intersection of the multiple independent HCC DNA methylation biomarker sets. Horizontal bars represent the total number of unique features in the respective signature. Vertical bars represent the number of features unique to the dataset, or in case of multiple signatures the number of overlapping features. The signatures selected to draw the barplots are identified with black circles. b) evaluation of the leave-one-out cross-validation procedure in the Train & Test dataset using the Recall, Precision, Specificity, Mathew’s Correlation Coefficient (MCC) and the balanced accuracy metrics. Each HCC DNA methylation feature set is coloured differently as in c). c) Precision-recall curves obtained by each feature set using the confidence scores of each sample belonging to the HCC class. Confidence scores are proportional to the sample distance to the hyperplane. Optimal F1-scores along the curves are marked with a “x”. Number of features in each dataset overlapping with the Train & Test dataset is specified in the label, as well as the area under the precision-recall curve and the MCC. Random precision is represented as a dashed horizontal line. d) confusion matrices of the predictions of each HCC DNA methylation biomarker set. e) similar to Figure 4b, but instead a 5-fold cross-validation procedure is used to calculate precision and recall rates using a logistic and ridge regression classifier ensemble.


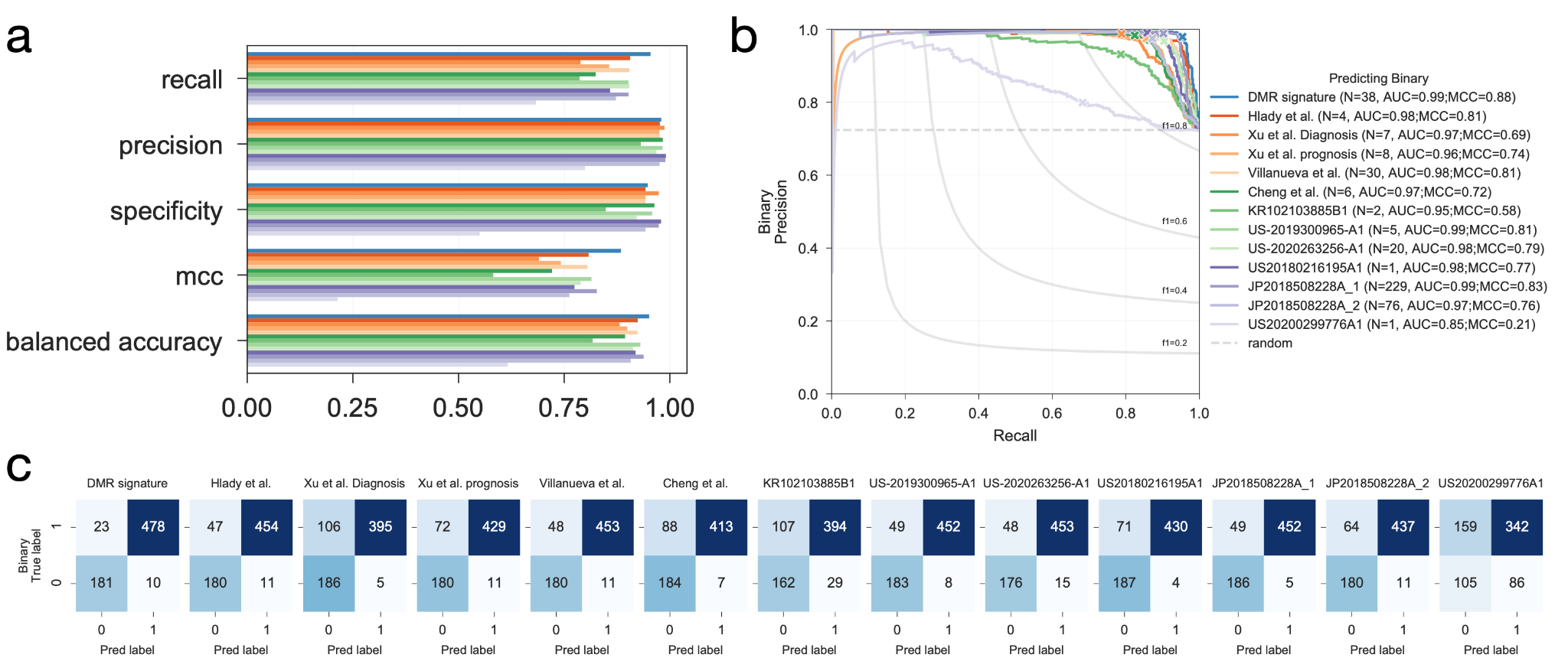


**Supplementary Figure 5. Validation dataset prediction performance.** a) evaluation of the Validation dataset using an ensemble of logistic and linear ridge classification models trained with the Train & Test dataset. Recall, Precision, Specificity, Mathew’s Correlation Coefficient and balanced accuracy metrics are presented. b) Precision-recall curves obtained by each feature set using the confidence scores of each sample belonging to the HCC class. Confidence scores are proportional to the sample distance to the hyperplane. Optimal F1-scores along the curves are marked with a “x”. Number of features in each dataset overlapping with the Validation dataset is specified in the label, as well as the area under the precision-recall curve and the MCC. Random precision is represented as a dashed horizontal line. c) confusion matrices of the predictions of each HCC DNA methylation biomarker set.


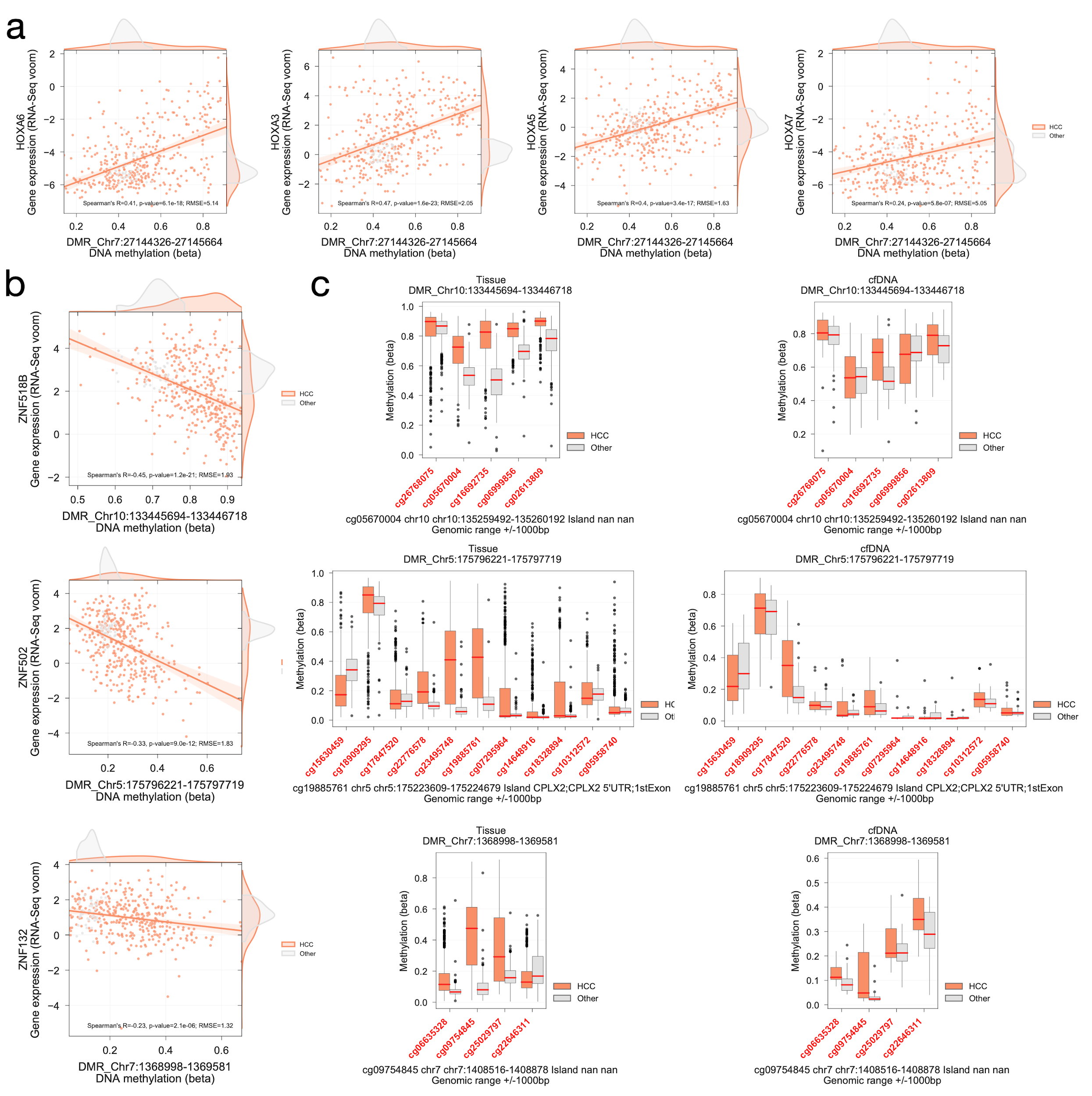


**Supplementary Figure 6. DNA methylation distribution of representative top CpG clusters predictive of HCC biomarkers.** a) significant associations between DMR Chr7:27144326-27145664 and gene expression of multiple HOXA members. b) similar to a), but instead showing significant associations between multiple Zinc Finger Proteins and DMRs Chr10:133445694-133446718, Chr5:175796221-175797719 and Chr7:1368998-1369581. c) DNA methylation (beta) of CpG sites contained in a range of 1,000 base-pairs of the DMRs presented in b). Genomic information of the location of the DMR to genes and CpG islands is provided below. Left panel shows the distribution of the DNA methylation in the tissue samples of the Test & Train datasets, while the right panel shows the distribution in the cfDNA samples of the same CpG sites. CpG sites are sorted in ascending order according to their genomic location. In red are CpG sites that are contained in the DMR.


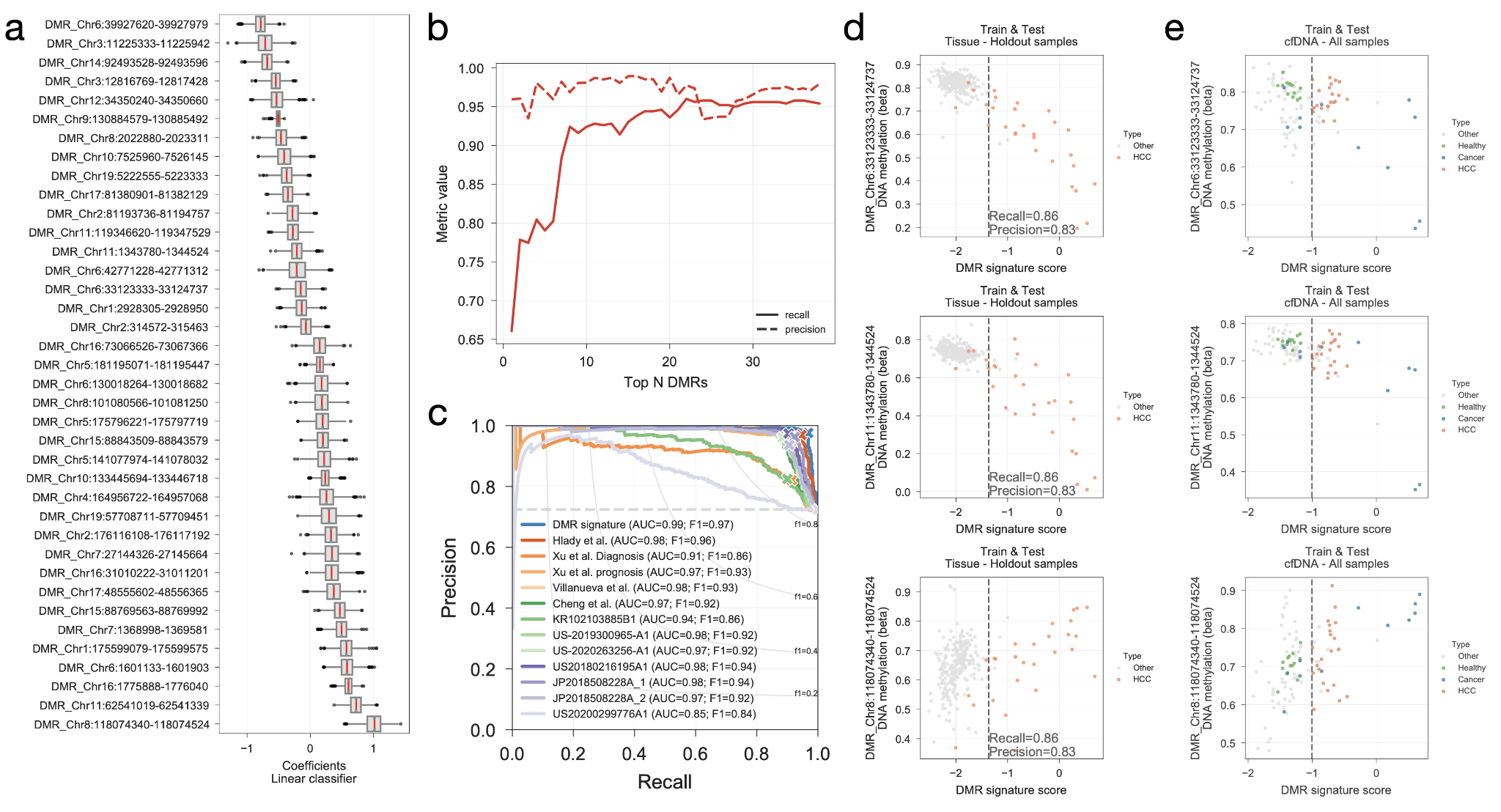


**Supplementary Figure 7. HCC DNA methylation risk score.** a) distributions of DMR coefficients across the 1,000 permutations of the balanced datasets. b) Recall and precision metrics of the predicted HCC labels in the Validation dataset using an iterative addition of the top N DMRs ranked descendingly according to their absolute mean coefficients reported in a). Train & Test dataset is used to train a cross-validated ensemble of logistic and linear classifiers to predict HCC samples in the Validation dataset. c) Precision-recall curves of the Validation samples calculated using linear risk score estimated from the mean coefficients obtained in the 1,000 permutation analysis. d) HCC DMR signature score calculated for all the samples in the Train & test dataset which were not used for the identification of the DMR signature and score nor their weights. DMR signature score plotted against three representative HCC DNA methylation biomarkers. HCC classification threshold is represented by a dashed vertical line and precision and recall rates are reported. e) Similar to d), instead only cfDNA samples are utilised and cfDNA samples from patients with other cancers (marked as blue and labeled as “Cancer”) are also considered as a positive event. cfDNA samples from healthy controls are marked in green (“Healthy”).


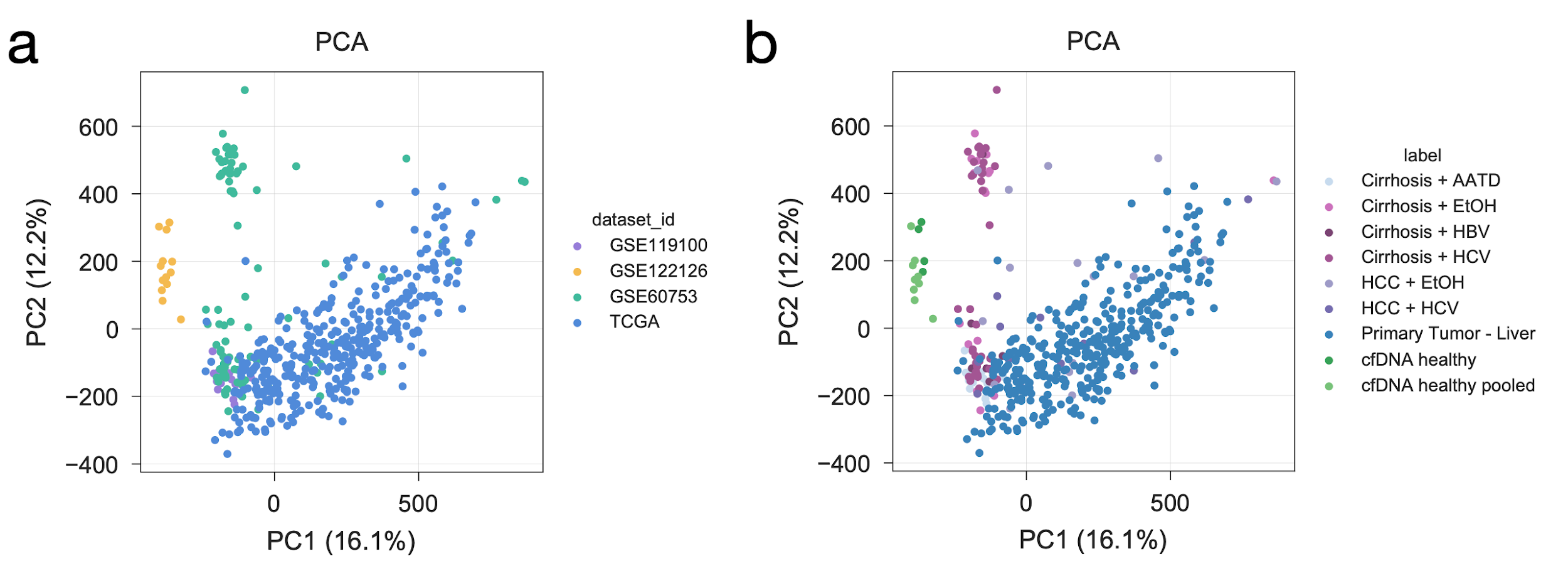


**Supplementary Figure 8. Train and Test PCA analysis.** First and second principal component of the Train & Test dataset samples coloured by a) original dataset, and b) sample type.

## **Supplementary Files**

**Supplementary Table 1.** List of samples and original studies that comprise the Train & Test and Validation datasets.

**Supplementary Table 2.** DMRs present in the optimal LinearSVC across the leave-one-out cross-validation procedure, frequency and effect sizes are reported.

**Supplementary Table 3.** Genomic information for all CpG sites mapping to the top 38 DMRs.

**Supplementary Table 4.** Significant CpG cluster - Gene expression associations.

**Supplementary Table 5.** HCC linear risk scores for the Test & Train and Validation datasets.

**Supplementary File 1.** Analyses source code.
